# Synthesis and Oxidative Degradation of Leucine-Based Poly(diacylhydrazine)

**DOI:** 10.3390/polym16091222

**Published:** 2024-04-27

**Authors:** Kanda Wongwailikhit, Ratha Suwannakeeree, Nobuhiro Kihara

**Affiliations:** 1 Department of Chemistry, Faculty of Science, Rangsit University, Phaholyothin Road, Lak-Hok, Pathum Thani 12000, Thailand; lionkicksheep@hotmail.com; 2 Department of Science, Faculty of Science, Kanagawa University, Rokkakubashi, Kanagawa-ku, Yokohama 221-8686, Japan

**Keywords:** oxidatively degradable polymers, poly(diacylhydrazine), sodium hypochlorite, on-demand degradation, leucin-based polymer

## Abstract

Diacylhydrazine is thermally and chemically stable, and it remains inert to oxygen even at high temperatures. However, it is rapidly oxidized by sodium hypochlorite, leading to its decomposition into carboxylic acid and nitrogen gas. In the synthesis of a novel poly(diacylhydrazine) as an oxidatively degradable polymer, L-leucine methyl ester is acylated by terephthaloyl chloride. Subsequent hydrazination yields a bishydrazide monomer. The oxidative coupling polymerization of this monomer produces poly(diacylhydrazine). The molecular structures of the products are confirmed by an ^1^H NMR analysis. A polymodal molecular weight distribution and a large polydispersity index are observed by GPC in all cases. A 10% weight loss temperature is noted at 286 °C in air by TGA. The obtained polymer is not oxidized by oxygen. No glass transition is observed below the decomposition temperature. Upon the treatment of the poly(diacylhydrazine) with sodium hypochlorite solution, decomposition occurs rapidly, resulting in monomeric carboxylic acid and nitrogen gas. The L-leucine-based poly(diacylhydrazine) serves as a novel on-demand degradable polymer with high levels of thermal and chemical stability during usage.

## 1. Introduction

**Scheme 1:**
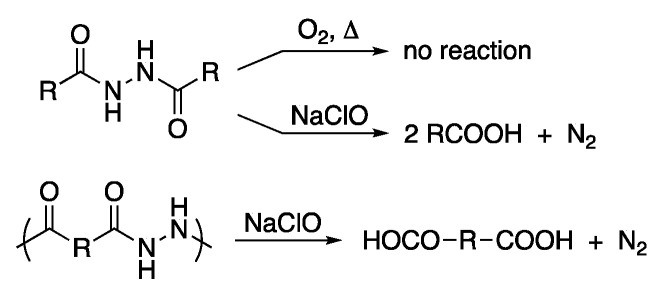
Oxidative degradation of diacylhydrazine.

Degradable polymers have attracted much attention for reducing polymer waste and facilitating polymer and/or material recycling [1,2,3,4]. However, their high level of stability during use generally conflicts with their high level of degradability after use [5]. An on-demand degradable polymer with high levels of thermal and chemical stability is strongly desired [6,7]. Poly(diacylhydrazine) represents a specific class of polyamide, bearing a doubly acylated hydrazine (-CO-NH-NH-CO-) group [8]. Although poly(diacylhydrazine) exhibits high levels of chemical and thermal stability similar to those of polyamides [9], it is rapidly degraded by specific oxidants such as sodium hypochlorite, while remaining inert to natural oxidants like oxygen, even at high temperatures (Scheme 1) [10,11,12,13,14]. Based on the oxidative degradability of diacylhydrazine, several applications including an oxidatively degradable superabsorbent polymer [15] were developed.

**Scheme 2:**
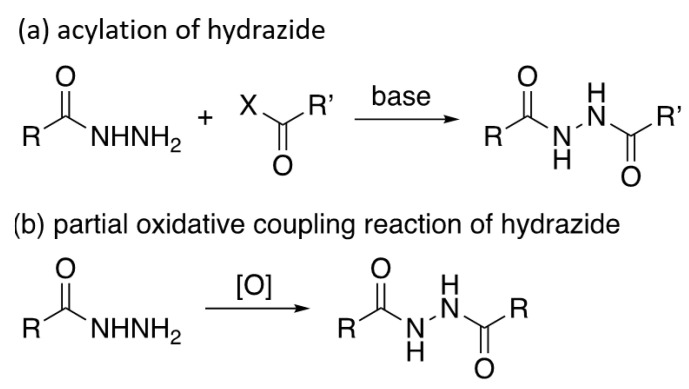
Synthesis of diacylhydrazine.

Poly(diacylhydrazine) can be effectively synthesized from hydrazide, not only through the acylation of acid halides (Scheme 2a) but also via partial oxidative coupling reactions (Scheme 2b) [16,17,18,19,20]. While the use of various dicarboxylic acids as reagents to react with hydrazine for synthesizing dihydrazide as the monomer is common [21,22,23], numerous efforts have been made to innovatively modify dicarboxylic acids with amino acids. This leads to the derivation of alternative dicarboxylic acids that enhance applications based on their aqueous solubility and biocompatibility [24]. This modification not only broadens the application spectrum of these novel materials but also aligns with our commitment to environmental sustainability [25]. Our work underscores the potential of amino-acid-based polymers in advancing both the functional versatility and eco-friendliness of degradable polymer applications.

In this study, L-leucine, a naturally occurring amino acid, was used to modify terephthalic acid, one of the most common dicarboxylic acids. The existence of a peptide bond in the skeleton and an isobutyl side chain in leucine is expected to enhance the flexibility of poly(diacylhydrazine) [26,27]. By using an optically active natural amino acid, future applications for it as a chiral polymer are also anticipated.

## 2. Materials and Methods

### 2.1. Chemicals and Instruments

All chemicals were reagent grade and were used without further purification except for dichloromethane and triethylamine, which were used after distillation over calcium hydride.

NMR spectra were recorded on a JEOL (Tokyo, Japan) JNM-ECA400 or JNM-ECZ600 FT-NMR spectrometer using residual solvent peak (2.50 ppm for DMSO-d_6_ and 7.26 ppm for CDCl_3_) as a reference. Thermogravimetric analysis (TGA) and differential thermal analysis (DTA) were performed on a Rigaku (Tokyo, Japan) TG8120 instrument. Differential scanning calorimetry (DSC) was performed on a Rigaku (Tokyo, Japan) DSC8230 instrument. Gel permeation chromatography (GPC) was performed using a Shimadzu (Kyoto, Japan) LC-10AT system with a Polymer Laboratories (CA, USA) PolyPore column placed in a Shimadzu (Kyoto, Japan) CTO-10A oven equipped with Shimadzu (Kyoto, Japan) RID-10A and Shimadzu (Kyoto, Japan) SPD-10A detectors.

### 2.2. Synthesis of Terephthaloyl Bis(L-Leucine Methyl Ester) ***2***

To a mixture of L-leucine methyl ester hydrochloride **3** (8.0853 g, 44.5 mmol) and terephthaloyl chloride **4** (4.1370 g, 20.4 mmol) in dichloromethane (100 mL), triethylamine (12.50 mL, 89.7 mmol) was added at 0 °C in the period of 30 min, and the mixture was stirred at 25 °C for 24 h. The reaction mixture was washed by 1 M hydrochloric acid followed by brine and dried over anhydrous magnesium sulfate, and the solvent was evaporated in vacuo. The crude white solid was recrystallized from toluene to obtain terephthaloyl bis(L-leucine methyl ester) **2** (4.6843 g, 55%) as a white crystal.

**^1^**H NMR (DMSO-d_6_, 400 MHz): d 8.87 (d, *J* = 7.6 Hz, 2H, NH), 7.97 (s, 4H, Ar), 4.56–4.47 (m, 2H, CH), 3.65 (s, 6H, CH_3_), 1.85–1.54 (m, 6H, CH_2_ and CH), 0.93 (d, *J* = 6.5 Hz, 6H, CH_3_), and 0.89 (d, *J* = 6.5 Hz, 6H, CH_3_) ppm.

### 2.3. Synthesis of Dihydrazide Monomer ***1***

A solution of terephthaloyl bis(L-leucine methyl ester) **2** (4.5899 g, 10.9 mmol) and hydrazine monohydrate (2.4459 g, 48.9 mmol) in 30 mL ethanol was refluxed for 24 h. The precipitate was collected by suction filtration, washed with ethanol, and dried in vacuo. Crude product was recrystallized from ethanol to obtain monomer **1** (3.1781 g, 69%) as a white crystal.

**^1^**H-NMR (DMSO-d_6_, 600 MHz): d 9.25 (s, 2H, NH), 8.55 (d, *J* = 8.1 Hz, 2H, NH), 7.95 (s, 4H, Ar), 4.54–4.47 (m, 2H, CH), 4.24 (br, 4H, NH_2_), 1.74–1.47 (m, 6H, CH_2_ and CH), 0.91 (d, *J* = 6.5 Hz, 6H, CH_3_), and 0.87 (d, *J* = 6.5 Hz, 6H, CH_3_) ppm.

### 2.4. Synthesis of Poly(diacylhydrazine) ***5***: Oxidative Coupling Polymerization of Dihydrazide Monomer **1**

To a solution of dihydrazide monomer **1**, the oxidant powder was added portion by portion (see dosage and conditions in Table 1). After the mixture was stirred for the mentioned period at ambient temperature, the reaction mixture was poured into 700 mL of methanol. The precipitate was collected by filtration, washed with methanol followed by water, and dried in vacuo to afford poly(diacylhydrazine) **5**.

^1^H NMR (DMSO-d_6_, 600 MHz): d 10.05 (br, 2H, NH), 8.62 (br, 2H, NH), 7.95 (br, 4H, Ar), 4.60 (br, 2H, CH), 1.79 (br, 4H, CH_2_), 1.59 (br, 2H, CH), 0.92 (d, *J* = 4.6 Hz, 6H, CH_3_), and 0.88 (d, *J* = 4.0 Hz, 6H, CH_3_) ppm.

### 2.5. Oxidative Degradation of Poly(diacylhydrazine) ***5***

A 5% sodium hypochlorite solution (10 mL) was poured on a powder of poly(diacylhydrazine) **5** (172.7 mg). Evolution of nitrogen gas promptly occurred and continued for 30 min to obtain clear solution. The reaction mixture was acidified with 3 M hydrochloric acid. After gas evolution ceased, precipitate was collected by suction filtration, washed with water, and dried in vacuo to obtain terephthaloyl bis(L-leucine) **6** (38.4 mg, 10%) as a black powder.

^1^H NMR (DMSO-d_6_, 600 MHz): d 12.7 (br, 2H, OH), 8.74 (d, *J* = 7.1 Hz, 2H, NH), 7.97 (s, 4H, Ar), 4.49–4.41 (m, 2H, CH), 1.83–1.52 (m, 6H, CH_2_ and CH), 0.93 (d, *J* = 6.4 Hz, 6H, CH_3_), and 0.88 (d, *J* = 6.3 Hz, 6H, CH_3_) ppm.

## 3. Results

### 3.1. Synthesis of Monomer ***1***

**Scheme 3:**
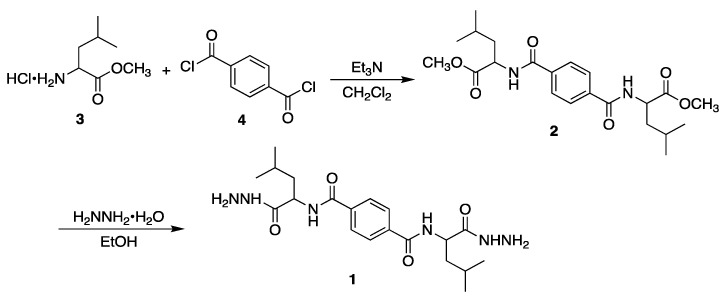
Synthesis of monomer.

**Figure 1:**
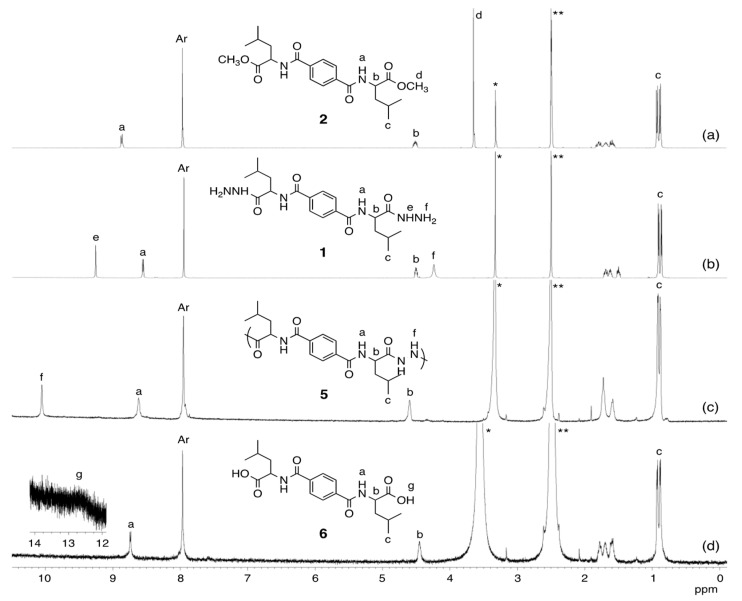
^1^H NMR spectra of (**a**) terephthaloyl bis(L-leucine methyl ester) **2** (DMSO-d_6_, 400 MHz), (**b**) dihydrazide monomer **1** (DMSO-d_6_, 600 MHz), (**c**) poly(diacylhydrazine) **5** (DMSO-d_6_, 600 MHz), and (**d**) oxidative degradation product **6** (DMSO-d_6_, 600 MHz) with low field region (inset). A signal with * denotes water, and a signal with ** denotes solvent peak.

Dihydrazide monomer **1** was synthesized according to the literature [28] with some modifications, as shown in Scheme 3. Terephthaloyl bis(L-leucine methyl ester) **2** was prepared by the acylation of L-leucine methyl ester hydrochloride **3** with terephthaloyl chloride **4**. The hydrazination of **2** afforded hydrazide monomer **1**. Both reactions proceeded smoothly, and monomer **1** could be obtained at gram scale without using a chromatographical purification procedure. The ^1^H NMR spectra of the products are shown in Figure 1.

Figure 1a clearly shows the structure of **2**. Two doublets at 0.93 and 0.89 ppm are characteristic of the non-equivalent methyl groups in leucine. Amide protons were observed at 8.87 ppm as a doublet. After hydrazination, the methyl ester in **2** observed at 3.65 ppm disappeared while signals for the hydrazide group in **1** appeared at 9.25 (NH) and 4.24 (NH_2_) ppm (Figure 1b).

### 3.2. Synthesis of Polymer

The oxidative coupling polymerization procedure was employed for the synthesis of poly(diacylhydrazine) because of its simple operation [28]. Thus, the partial oxidation of dihydrazide **1** was carried out using Oxone^®^ [18] (2KHSO_5_·K_2_SO_4_·KHSO_4_) and diacetoxyiodobenzene [17] (PhI(OAc)_2_), respectively. The reaction conditions and the results of the polymerization are summarized in Table 1. The ^1^H NMR spectrum of the polymer obtained (run 1) is shown in Figure 1c. The NH_2_ protons in **1** observed at 4.24 ppm disappeared, and the hydrazide NH proton observed at 9.25 ppm shifted down-field (10.02 ppm), while the signals of L-leucine, including two characteristic non-equivalent methyl groups (0.92 and 0.88 ppm) and an amide-NH group (8.62 ppm), remained. These observations indicate that poly(diacylhydrazine) **5** was obtained via the selective oxidation of the hydrazide group without the oxidation of the L-leucine amide moiety.

In each experiment, the yield of the polymer was moderate to high. When the oxidative coupling polymerization was carried out using Oxone^®^ as the oxidant, a white polymer was obtained, while a pink polymer was obtained when oxidized with PhI(OAc)_2_. This variation in color suggests the occurrence of a side reaction when PhI(OAc)_2_ was used as the oxidant, although no significant difference was observed in the ^1^H NMR spectra.

Poly(diacylhydrazine) **5** was soluble in aprotic polar solvents, such as DMF, DMAc, and DMSO, and was insoluble in water and common organic solvents, such as THF and chloroform.

**Figure 2:**
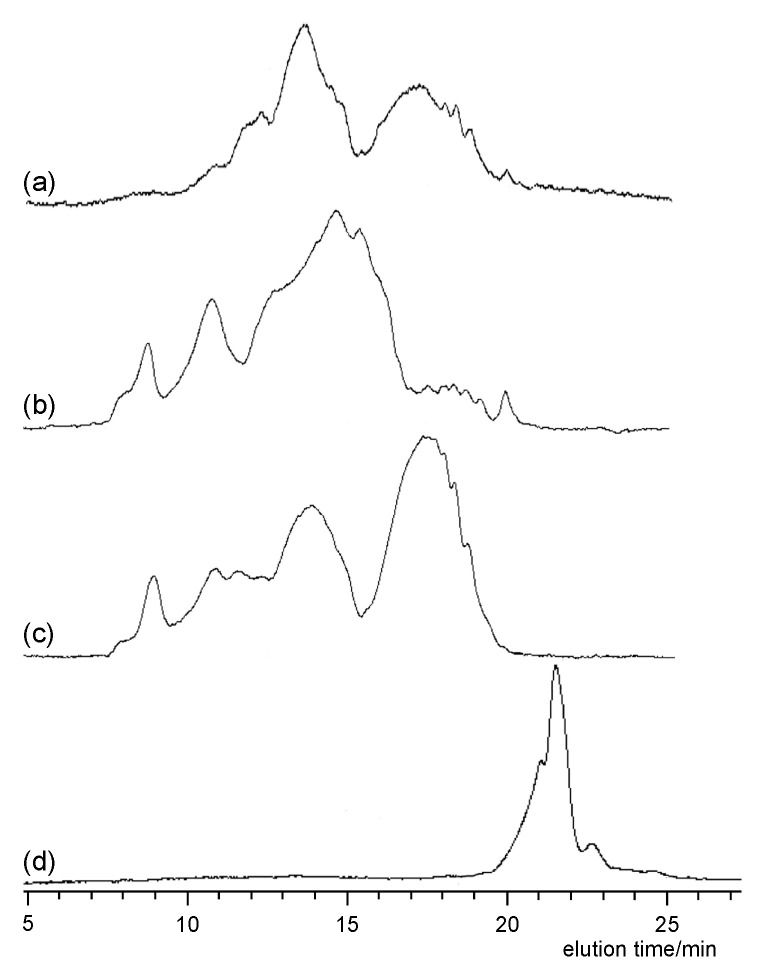
GPC profiles of poly(diacylhydrazine) **5** obtained by the oxidative coupling polymerization of hydrazide monomer **1**. (**a**) Run 1, (**b**) run 2, and (**c**) run 3 in Table 1 and (**d**) oxidative degradation product.

The gel permeation chromatography (GPC) profiles are shown in Figure 2. Polymer **5** showed a polymodal molecular weight distribution, and the M_w_/M_n_ value estimated from GPC was far larger than that expected for polycondensation (2.0) in every case. Similar results were observed in the previous work [28]. At the present time, the reason for the large M_w_/M_n_ values is not clear, although there are two possibilities. One is the presence of a crosslinked structure in the polymer structure. However, there is no spectroscopic evidence or sign of the crosslinked structure. The other is the aggregation of the polymer chain because of the strong hydrogen bonding ability of the diacylhydrazine moiety. In this case, the M_w_ value was reflected by the highly aggregated part, and the M_n_ value was reflected by the less aggregated part. Therefore, the large M_w_/M_n_ values were observed. Since poly(diacylhydrazine) **5** is soluble in DMF even though a very large M_w_/M_n_ value was observed, we had no difficulty in its application.

### 3.3. Thermal Properties of Poly(diacylhydrazine) ***5***

**Figure 3:**
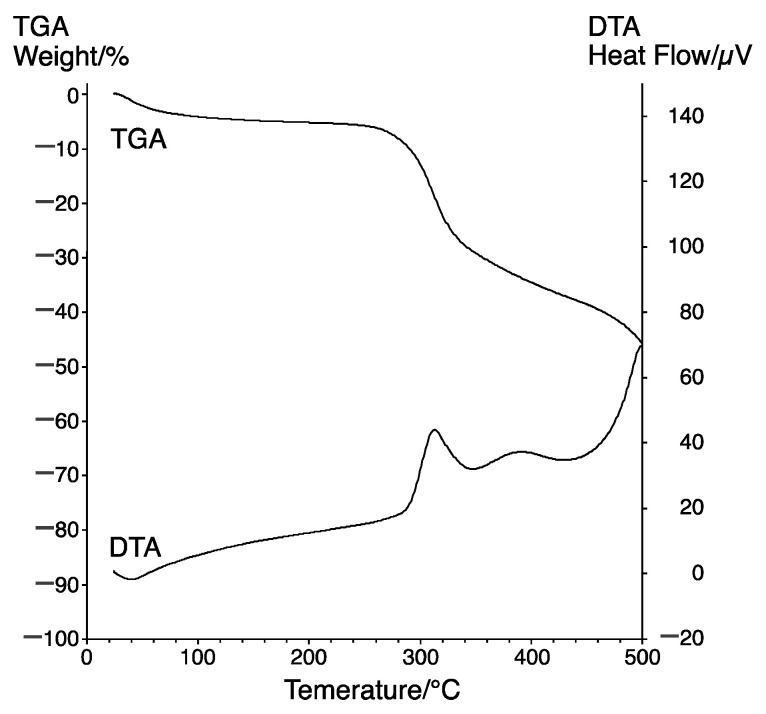
TGA-DTA profile of poly(diacylhydrazine) **5** (10 K/min, in air). In the DTA analysis, positive heat flow is exothermic.

A thermogravimetric analysis (TGA) and differential thermal analysis (DTA) were performed in air to examine the thermal properties of **5** as shown in Figure 3. Upon heating, ca. a 5% weight loss was observed below 100 °C due to the loss of absorbed water. It was assumed that 0.5 molecule of water per repeating unit was absorbed in **5**, as the calculated weight loss is 4.43%. A 10% weight loss was observed at 286 °C. Thus, **5** is thermally stable and does not suffer oxidative degradation by oxygen in air even at a high temperature [14,28]. In our careful differential scanning calorimetry (DSC) analyses, **5** does not exhibit a glass transition temperature (T_g_) below 250 °C. It is very likely that the hypothetical T_g_ of **5** is higher than its thermal decomposition temperature, supporting the high thermal stability of **5**.

### 3.4. Oxidative Degradation of Poly(diacylhydrazine) ***5***

When an aqueous solution of sodium hypochlorite was poured on the powder of poly(diacylhydrazine) **5**, a nitrogen gas bubble occurred vigorously, and a clear solution was obtained within a few minutes. Since **5** is insoluble in water, the oxidative degradation of **5** was evident. The GPC profile of the resulting solution is shown in Figure 2d, indicating that the polymeric fraction completely disappeared, and a low-molecular-weight compound was formed.

**Scheme 4:**
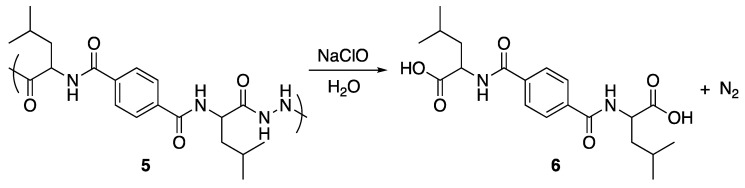
The oxidative degradation of poly(diacylhydrazine) **5** using sodium hypochlorite solution.

When the solution was acidified by 3 M hydrochloric acid, a dark-brown precipitate was obtained. The ^1^H NMR spectrum of this product is shown in Figure 1d. The signal of the diacylhydrazine group observed at 10.02 ppm for **5** disappeared, while the signals for the L-leucine terephthalamide skeleton remained intact, including its characteristic two non-equivalent methyl groups (0.93 and 0.88 ppm). Further, a broad signal for the carboxylic acid group was observed around 12.7 ppm. Thus, the diacylhydrazine group was selectively oxidized in the presence of the amide by the sodium hypochlorite to form carboxylic acid **6** and nitrogen gas, as depicted in Scheme 4.

## 4. Conclusions

This study demonstrated the successful synthesis of a novel poly(diacylhydrazine), poly(diacylhydrazine) **5**, from terephthaloyl chloride, L-leucine methyl ester, and hydrazine monohydrate using the oxidative coupling polymerization process. Although polymer **5** was thermally and chemically stable, its oxidative degradation proceeded rapidly using a sodium hypochlorite solution. It should be noted that the degradation products, carboxylic acid and nitrogen gas, are safe compounds, and carboxylic acid **6** can be used as a starting material for the synthesis of **5**. Further, the sodium hypochlorite solution is easily available as a common household bleaching agent. Since various amino acids are expected to be used instead of L-leucine for the synthesis of corresponding poly(diacylhydrazine)s, various on-demand degradable polymers are expected. Further applications of this novel poly(diacylhydrazine) are in progress.

## Figures and Tables

**Table 1:** The reaction conditions and the results of oxidative coupling polymerization of **1**.

Run	1 (g)	Oxidant (g, eq)	Solvent (mL)	Time (h)	Yield ^a^ (%)	M_n_^b^ (M_w_*/*M_n_^b^)
1	1.0111	Oxone^®^ (2.9724, 2.0)	NMP/CH_3_CN/H_2_O (10/10/7)	48	74	4600 (98)
2	0.5062	Oxone^®^ (1.4831, 2.0)	NMP/DMAc/H_2_O (10/10/8)	48	57	6700 (12,000)
3	0.4935	PhI(OAc)_2_ (0.6656, 0.88)	NMP (5)	24	74	5500 (7300)

^a^ Methanol-insoluble part. ^b^ Estimated by GPC (DMF, based on polystyrene standards).

## Data Availability

The data presented in this study are available upon request from the corresponding author. The data are not publicly available due to restrictions from the funders.
